# Editorial: HIV and Illicit Drugs of Abuse

**DOI:** 10.3389/fmicb.2016.00221

**Published:** 2016-03-10

**Authors:** Venkata S. R. Atluri

**Affiliations:** Department of Immunology, Institute of NeuroImmune Pharmacology, Herbert Wertheim College of Medicine, Florida International UniversityMiami, FL, USA

**Keywords:** HIV-1, AIDS, HIV-1 Tat, HIV-1 gp120, Drug abuse, NeuroAIDS, HAND

Drugs of abuse plays a major role in increasing the risk of HIV transmission, disease progression, and less adherence to the antiretroviral therapy, which significantly contribute for morbidity and mortality of the HIV infected patients (Anthony et al., 1991; Arnsten et al., 2002). While injection drug use is the second most common route for the HIV transmission, alcohol consumption, smoking, inhaling, and ingesting drugs such as heroin, morphine, crack cocaine, methamphetamine (METH) will increase the risk for obtaining HIV infection (Friedland and Vlahov, 2011). Heroin is the most commonly used illicit drug in HIV patients followed by stimulants such as cocaine and METH (AIDSinfo, 2012). Drug abuse is one of the risk factors for severe neurocognitive dysfunctions in HIV-positive individuals (Nath et al., 2002). Therefore, drugs of abuse and their role on inducing HIV pathogenesis/neuropathogenesis is an urgent need to study for the development of potential therapeutics for HIV infected drug abuser. In this special issue, Garin et al. reported that recreational drug use is higher in HIV infected persons than the general population and cannabis is the highly used recreational drug in the Europe followed by cocaine, amphetamines and ecstasy.

Opioid abuse increases the HIV disease progression by effecting host immune function, promoting the virus entry into the immune cells and replication and it also causes severe neurocognitive disorders by inducing the neuro-inflammation (Roy et al., 2011; Smith et al., 2014). While gut microbiota helps in regulating immune homeostasis, both HIV and opioids are known to disrupt gut homeostasis, gut immunity, and microbial translocation that may lead to the accelerated HIV disease progression. In this context, Meng et al. reviewed the mechanisms of opioid induced HIV disease progression by disrupting the gut homeostasis. As pathological pain is more common in 50% of HIV/AIDS patients, HIV-infected opioid abusers reported to show severe neuropathology than HIV-infected non-drug users (Bell et al., 1998, 2006; Smith et al., 2014). Liu et al. reviewed potential mechanisms that induce neuropathic pain in HIV and opioids interaction. While heroin has been reported to increase the HIV infection in macrophages by inhibiting the HIV restriction miRNAs (miRNA-28, miRNA-125b, miRNA-150, and miRNA-382), naltrexone (opioid-receptor antagonists) reported to recover the expression of these miRNAs (Wang et al.). Also, Lan et al. discussed about the association of Apolipoprotein L1 (APOL1) variants (G1 and G2 alleles) in Heroin-associated Nephropathy (HAN) and Human Immunodeficiency Virus associated Nephropathy (HIVAN) in African Americans which opens an interesting point to explore the onset of focal and segmental glomerulosclerosis (FSGS) in HIV infected heroin abusers in African Americans. In the post-mortem brain tissues of HIV-infected subjects with neurocognitive impairment (NCI) ± HIV encephalitis (HIVE), Dever et al. reported the dysregulation of autophagy genes and proteins; and in *in vitro*, supernatant from HIV-1-infected microglia/HIV-1 Tat protein in combination with morphine reported to alter autophagic activity and reduced dendrite length. Morphine in combination with HIV-1 gp120 has been reported to increase oxidative stress, DNA damage, and subsequently affecting cell cycle process (Samikkannu et al.).

Cocaine is the second most commonly abusing drug in US and cocaine abuse in HIV infected patients reported for rapid progression to AIDS and are prone to develop severe form of neurocognitive disorders (Avants et al., 1997; Baldwin et al., 1998; Zenon et al., 2014). In this special issue, Bertrand et al. reported that R-Equol, S-Equol (derivatives of the soy isoflavone, daidzein) reduces the neurotoxic effects (reduced dendritic synapses) of cocaine in combination with HIV-1 Tat in an estrogen receptor beta dependent manner. Roy et al. reported the reduced levels of DJ1 protein (a gene linked to autosomal recessive early-onset Parkinson's disease) and associated increased ROS production in HIV exposed neuronal cells in combination with cocaine. Also two comprehensive review articles focused on the effect of cocaine abuse on HIV pathogenesis (Dash et al.) and role in progression to HIV-1 associated neurocognitive disorders (Dahal et al.).

In North America, methamphetamine is the widely used recreational drug especially in men who have sex with men (MSM) infected with HIV (Colfax and Shoptaw, 2005). In this special issue, Bortell et al. reported that mononuclear cells isolated from SIV infected METH treated brain has significantly upregulated IL2RG and its ligand cytokine (IL15, IL15RA) levels compared to the control and SIV alone infected animals which can aggravate the neuroinflammation. Borgmann and Ghorpade have contributed a comprehensive review on alterations in astrocyte intrasignaling pathways, gene expression, and function in the presence of HIV and its proteins in combination with METH that contributes to the neuroinflammation and also focused on the therapeutics for astroglial activation and function. Mediouni et al. discussed about effect of METH and HIV-Tat on the development of HAND.

Alcohol use disorder is more common in the United States and in persons living with AIDS, rates of heavy drinking are even higher than those in the general population (Petry, 1999). Heavy alcohol consumption increases the risk of HIV transmission, higher viral load and lower adherence to ART (Baum et al., 2010). Agudelo et al. reported the higher HIV infectivity in alcohol treated monocyte derived dendritic cells (MDDC) and also observed differentially modulated HIV infection, altered MDDC endocytic function and cytokine production in combination with cannabinoids (THC and JWH-015).

In US, the prevalence of cigarette smoking range from 40 to 75% in HIV-infected individuals compared to 20% in general population (Pacek and Cioe, 2015). Nicotine is the active ingredient in tobacco and considered one of the most addictive drugs of all time and reported to dysregulate synaptic plasticity in HIV exposed neuronal cells (Atluri et al., 2014). On the other hand, direct injection of HIV-Tat into rat intra ventral tegmental area reported to attenuate nicotine-induced behavioral sensitization (Zhu et al.). Rao and Kumar reviewed the effect of smoking on HIV replication and role of polycyclic aromatic hydrocarbons (PAH-important constituents of cigarette smoking) and CYP1 enzymes (CYP1A1 and CYP1B1- activators of PAH) in HIV pathogenesis. Chinnapaiyan and Unwalla reviewed the mechanisms of mucociliary suppression in people living with HIV who are smokers or illicit drug abusers.

Also, this special issue include review articles on epigenetic alterations in drugs of abuse and HIV infection that affects Vitamin D receptors (Chandel et al.); role of miRNs during HIV infection and effect of drugs of abuse on the expression of miRNAs and its effect on HIV associated neurocognitive disorders (Pilakka-Kanthikeel and Nair); effect of HIV-1 Tat and drugs of abuse during HIV infection and their role in the development of NeuroAIDS (Maubert et al.); and anti-HIV effects of different natural compounds (Kurapati et al.).

## Author contributions

The author confirms being the sole contributor of this work and approved it for publication.

### Conflict of interest statement

The author declares that the research was conducted in the absence of any commercial or financial relationships that could be construed as a potential conflict of interest.
